# Evaluation of sphenopalatine ganglion blockade via intra oral route for the management of atypical trigeminal neuralgia

**DOI:** 10.1186/s40064-016-2612-8

**Published:** 2016-06-27

**Authors:** Ilker Coven, Ezher H. Dayısoylu

**Affiliations:** Department of Neurosurgery, Health University Medical Faculty, Training and Research Hospital, Meram Yeniyol No: 1, Meram/Konya, Turkey; Department of Oral and Maxillo-Facial Surgery, Faculty of Dentistry, Baskent University, Etimesgut/Ankara, Turkey

## Abstract

**Background:**

The sphenopalatine ganglion (SPG) may be involved in persistent idiopathic facial pain and unilateral headaches. The role of SPG blockade via intra oral route in the management of trigeminal neuralgia (TN) is worthy of study.

**Methods:**

In this retrospective study, patient records included patients with atypical TN (type 2) that persisted in spite of conservative treatment for at least 2 years, and an average pain intensity from the craniofacial region visual analogue scale (VAS) before examination. In group I the patients received carmapazepin 800 mg a day for at least 2 years. In group II 3 ml of local anesthetic agent consisting 2 ml bupivacaine and 1 ml prilocain in addition to 1 ml fentanyl, 0.5 ml betametasone disodium phosphate and 0.5 ml opaque was injected by the intraoral route. In this group, injection procedures were performed under local anesthesia with fluoroscopic guidance. The Kruskal–Wallis and Mann–Whitney U tests with Bonferroni correction were used for intergroup analysis. Age and sex differences were evaluated with one-way ANOVA and Fisher’s exact tests, respectively.

**Results:**

Significant differences were found between pre-op and 3rd day VAS values and also pre-op and 1st month VAS values. No significant differences were found between pre-op and 6th month VAS values.

**Conclusion:**

The SPG blockade improves the quality of life of patients and a minimally-invasive procedure to management of TN, when compared to other methods.

## Background

The International Headache Society recently defined strict clinical criteria for trigeminal neuralgia (TN) diagnosis (Headache Classification Committee of the International Headache Society 2013). Diagnosis of TN consists at least three attacks of unilateral facial pain occur fulfilling these criteria; (1) occurring in one or more trigeminal nerve division, (2) pain with at least three of the following four characteristics that are recurring paroxysmal attacks lasting from a fraction of a second to 2 min, severe intensity, electric shock-like shooting–stabbing or sharp in quality and precipitated by innocuous stimuli to the affected side of the face. In this new classification it has been proposed to differentiate TN into two types; type 1 (previously referred to as classic or typical TN), which is an idiopathic episodic pain with the previously reported clinical characteristics, lasting several seconds, with pain-free intervals between attacks and type 2, describing idiopathic trigeminal facial pain that is aching, throbbing, or burning pain for more than  % 50 of the time and is constant in nature with a minor component of sharp, episodic pain. It has been also theorized that TN type 1 can progress toward TN type 2 and that in this second type, the likehood of detecting a structural abnormality such as a tumor or a vascular malformation is higher (Eller et al. 2005). Sphenopalatine ganglion (SPG) may be involved in persistent idiopathic facial pain (PIFP) and unilateral headaches (Eller et al. 2005; Montano et al. 2015). A few motor nerves accompany the SPG sensory trunks. An irritation of the SPG motor root may; produce face and neck neuralgias by its connection with the facial nerve (FN), lesser occipital and cutaneous cervical nerves; account for disturbances in the eye and mandible region by its connections with the ciliary and otic ganglions and a variety of visceral symptoms by its connection with the vagus nerve; cause reflex otalgia by its connection with the tympanic plexus (Capra and Dessem 1992; Maarbjerg et al. 2014).

Recent research has highlighted the important role of the SPG in cerebrovascular autonomic physiology, in pathophysiology of cluster and migraine headaches, and in conditions of stroke and cerebral vasospasm (Piagkou et al. 2012; Day 2001; Broggi et al. 2005). The guidelines of American Academy of Neurology (AAN) and the European Federation of Neurological Societies (EFNS) suggest that carbamazepine (CBZ) and oxcarbazepine (OXC) are the first-line medical treatments for pain control in patients with TN (Cruccu et al. 2008; Attal et al. 2010). Although pharmacotherapy for the management of TN is widely accepted procedure, the side effects, need for high doses of medication and finally insensitivity to these agents leads to failure of this treatment modality (Besi et al. 2015). Therefore surgical and minimal invasive management strategies have been developed that also suggested by AAN–EFNS as an option for the patients refractory to drugs or have adverse reactions (Di Stefano et al. 2014). On the other hand success rate of these procedures varies considerably. SPG blockade via nasal route is a well described procedure for the management of TN. Bupivacaine, which is a long lasting local anesthetic agent, is applied via transnasal approach in this technique. However nasal route requires general anesthesia, endoscopic guidance and advanced technical skills. On the otherhand SPG blockade via intraoral route can be administered under local anesthesia even in office settings. Opioids are one of the most effective classes of medications available to treat both acute and chronic pain. Although its usage in these situations is described in literature, the effectiveness of local administration of opioids on the management of TN type 2 has not been studied before. In this study it was aimed to investigate the effectiveness of SPG blockade via intraoral route with bupivacaine and fentanyl combination for the management of TN type 2.

## Methods

In this retrospective study patients who had been referred to Baskent University Department of Oral and Maxillofacial Surgery and Neurosurgery for management of facial pain between October 2011 and February 2015 were included. The patient selection criteria obtained as International Headache Society clinical criteria for trigeminal neuralgia.

**Fig. 1:**
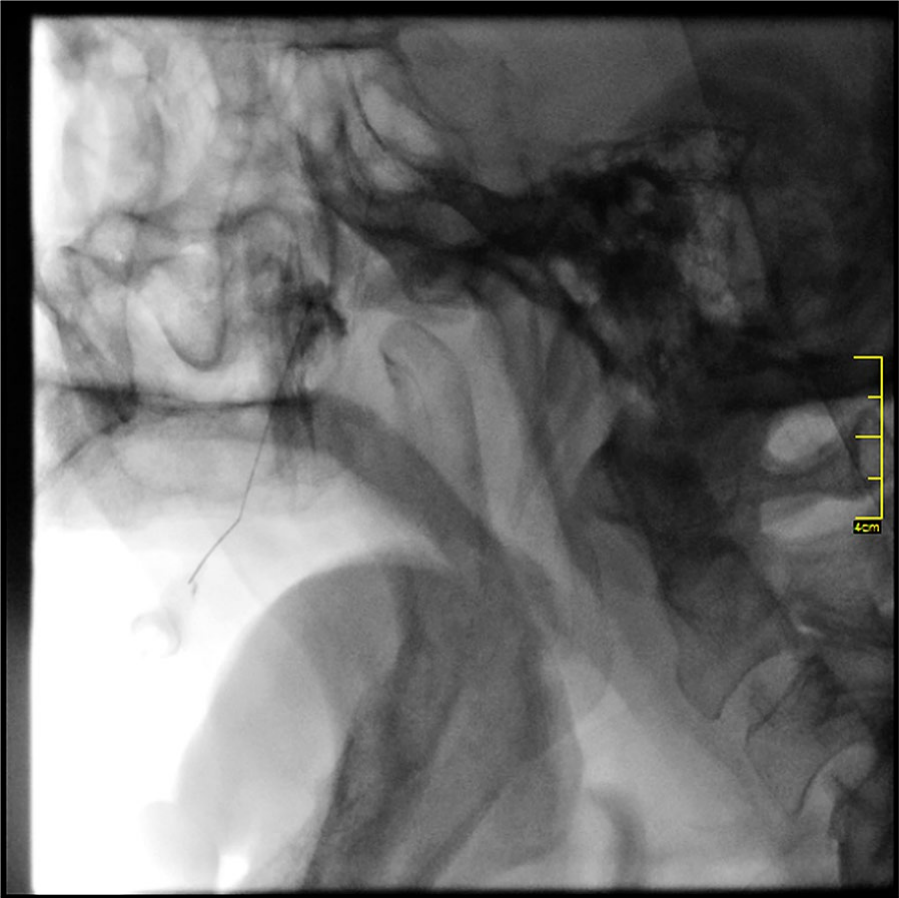
Once the tip of the needle reached the SPG under direct visualisation of the guidance, the mixture was injected

**Fig. 2:**
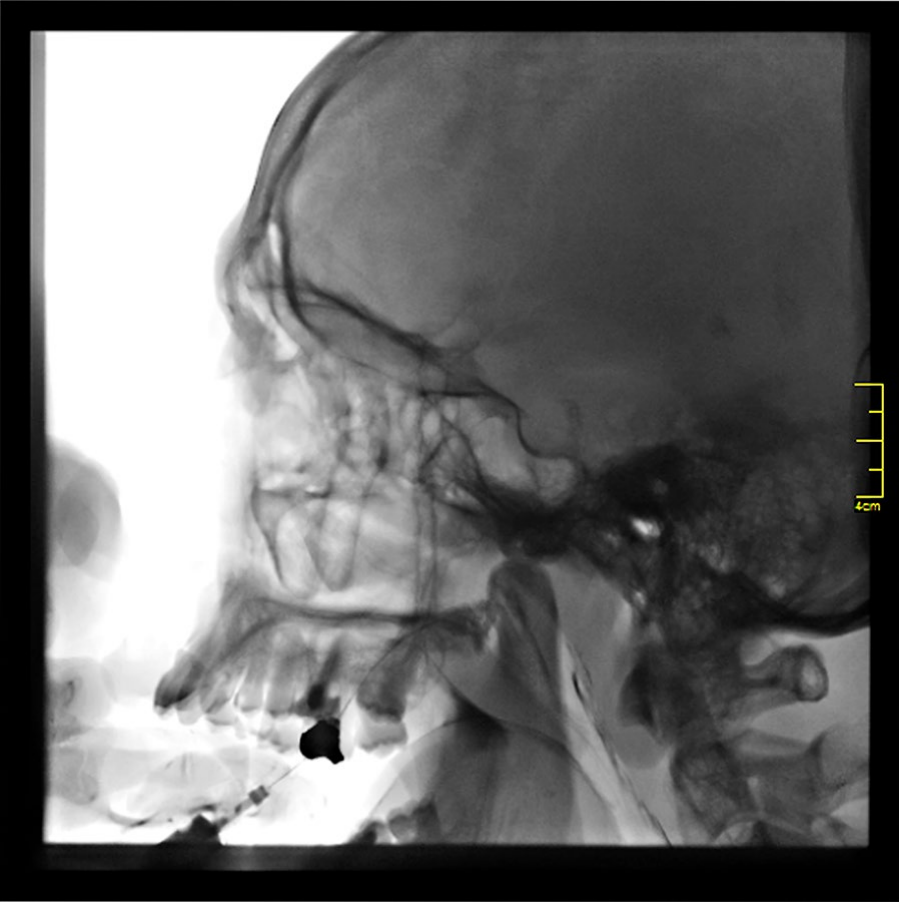
A-27-gauge dental needle was 60° tilted and inserted from 1 cm anterior and 1 cm medial position from the 3rd molar area. Needle was passed through from palatinum majus and it was forwarded to superior-posterior direction for an average 2 cm distance

A total number of 239 patient records were evaluated. The patients with other types of trigeminal neuralgia, cluster headache, patients with a history of previous surgical attempts to trigeminal neuralgia, diagnosed with multiple sclerosis or psycological disorders were excluded. In adddition patients who did not attend follow-up visits were also excluded. A-seventy-six patient (55 female, 21 male) who were referred for atypical trigeminal neuralgia between October 2011 and February 2015 were included. Group I consisted 35 female and 15 males (n = 50) and group II consisted 20 female and 6 males (n = 26) patients. Patient records were included that patients with atypical trigeminal neuralgia (TN type 2) persisted in spite of conservative treatment (oral carbamazepine 800 mg a day) for at least 2 years, and an average pain intensity from the craniofacial region of >30 mm on a 0–100 mm visual analogue scale (VAS) before examination (Cruccu et al. 2008). The VAS values were evaluated using a scale with two anchor points; zero being no pain and ten being the worst pain before the procedures. Patients with a minimum follow up time for 2 years were included whereas patients with other types of trigeminal neuralgia, cluster headache, operated for trigeminal neuralgia or psycological disorders were excluded (Cruccu et al. 2008). All patients were informed about the treatment modalities and informed consent form was obtained from the patients. Patients were evaluated at four different times as pre-operative, early immediate (3 day), immediate (1 month) and late (6 months) intervals. In group I the patients received carbamazepine (CBZ) 800 mg a day for at least 2 years. In group II 3 cc of local anesthetic agent consisting 2 cc bupivacaine and 1 cc prilocain in addition to 1 cc fentanyl, 1 cc betametasone disosium phosphate (celestone) and 1 cc opaque (omniscan) was injected from the intraoral route. In this group, injection procedures were performed under local anesthesia with fluoroscopic guidance. Patients were lay on the operating table and 1 cc of local anesthetic was applied to the palatal region of the second and third molar area. A-27-gauge dental needle was 60° tilted and inserted from 1 cm anterior and 1 cm medial position from the 3rd molar area. Needle was passed through from greater palatine foramen and it was forwarded to superior-posterior direction for an average 2 cm distance. Once the tip of the needle reached the SPG under direct visualisation of the guidance, the mixture was injected (Figs. 1, 2).

Outcome measures were pain intensity during daily activities such as eating and talking and also patient satisfaction quantified on VAS evaluation.

The Kruskal–Wallis and Mann–Whitney U tests with Bonferroni correction were used for intergroup analysis. Age and sex differences were evaluated with one-way ANOVA and Fisher’s exact tests, respectively. Variables were presented as the mean ± SE of the mean, and the differences were considered significant at p < 0.01. SPSS 17.0 was used for analysis (Statistical Package for the Social Sciences, version 17.0, SSPS Inc. Chicago, IL, USA).

## Results

Statistically significant differences were found between the groups regarding the sex of the patients (p < 0.01). Intra-group and inter-group analysis revealed significant female predilection for atypical trigeminal neuralgia. The age range of the patients was 27–82 (mean age: 54.7 ± 11.3) years. No significant differences found between the groups regarding the age of the patients (p = 0.82).

Medical therapy was recommended for 50 patients in group I. The overall mean VAS values decreased from 8.0 to 3.8 ± 1.29 (VAS max 10–VAS min 0). No significant differences were found between pre-op and 3rd day VAS values and also pre-op and 1st month VAS values (p > 0.05). On the other hand significant differences were found between pre-op and 6th month VAS values (p < 0.05).

SPG blockade was administered for 26 patients in group II. The overall mean VAS values decreased from 8.4 to 2.8 ± 1.03 (VAS max 10–VAS min 0). Significant differences were found between pre-op and 3rd day VAS values and also pre-op and 1st month VAS values (p < 0.05). On the other hand no significant differences were found between pre-op and 6th month VAS values (p > 0.05). In Group I, sleepiness was noted in three of 50 patients (0.6 %). In Group II, temporary nausea was noted in one of 26 patients (0.3 %). No other complications were noted during the study period.

Intergroup analysis showed time dependent significant differences between the groups. Although no significant differences were found in pre-treatment evaluation between the groups, significant differences were found between the groups I and II at 3rd day and 1st month VAS values (p < 0.01). On the other hand no significant differences were found between the groups I and II at 6th month VAS values (p > 0.05).

## Discussion

Trigeminal neuralgia (TN) is characterized by a unilateral condition with ultra-short stabbing pain located along one or more branches of the trigeminal nerve. The primary treatment choice for the TN consists of anti-epileptics. Carbamazepine (CBZ) and oxcarbazepine (OXC) are the frequently used agents; on the other hand, nervousness, sleepiness, depression, gum diseases, concentration difficulties have been noted in patients (Besi et al. 2015). In addition prolonged TN pain has been noted to increase anxiety disorders and cognitive impairments (Wu et al. 2015). On the other hand late resistance to OXC and CBZ in cases of prolonged usage has been also noted (Di Stefano et al. 2014). Therefore surgical approaches should be kept in mind as recommended by AAN–EFNS in refractory cases.

SPG has been identified as the first relay station of the autonomic fibers after emerging from the pons suggesting that it may be used therapeutically in autonomic imbalance situations and it may play a critical role as a vasodilator to protect the brain against ischemia in stroke or ischemia of migraine with aura (Burstein and Jakubowski 2005). In addition SPG as a major source for postganglionic parasympathetic fibers to the vascular beds of the cerebral hemispheres is involved in tone regulation of the cerebral vessels (Piagkou et al. 2012). Crucial nerve fibers of SPG carrying them trigeminal nerve (TGN) in various pain syndromes such as atypical facial pain, vasomotor rhinitis, eye disorders, play a role in pain due to herpes infections.

TGN’s parasympathetic nerves, petrosal and postganglionic fibers lacrimal, palatine and follow nasal structures (Haviv et al. 2015; Benoliel et al. 2015; Love and Coakham 2001). Sensitive fibrils of SPG associated with the maxillary nerve. Nucleus salivatorius from the parasympathetic nerve ganglia from preganglion geniculi with facial nerve fibers reach the ganglion pterygopalatina with major petrosus (Capra and Dessem 1992; Maarbjerg et al. 2014; Rusu et al. 2009).

As the SPG has an important role on different pain syndromes, SPG blockade has an important role on the management of TN type 2. Many attempts including the removal of SPG cells, known as sphenopalatine ganglioneurectomy or radiosurgical neuroablation techniques have been described in the literature with varying success rates (Cepero et al. 1987; Karas et al. 2008). Besides, minimal invasive techniques via transnasal or infrazygomatic approaches described for modulating the action of SPG with different success rates. Transnasal or also known as intranasal approach one of the common approaches for the blockade of SPG, but topical anesthetic diffusion with cotton swabs to the SPG is unpredictable and the blockade is not durable. In addition nose abnormalities and also blunt surgical access make the success of the procedure contradictory. As a solution endoscopic transnasal approach was described by Isherwood et al. but the procedure requires endoscopic instrumentation and devices (Isherwood and Ansell 2016). Infrazygomatic approach has been commonly performed blindly on the other hand, SPG block via intra oral route roads safer compared to other percutaneous intervention for the improvement of acute symptoms, it was quick and efficient (Sanders and Zuurmond 1997). Although intra oral route is the most direct way to reach to the SPG, it was claimed as difficult, uncertain, and occasionally impossible to reach by some authors (Piagkou et al. 2012). That is partially acceptable because earlier approaches tend reach to SPG in a blinded fashion and makes the attempt unpredictable. On the other hand in our study group all the interventions were performed under the fluoroscopic guidance, therefore the procedure was noted as safe and predictable. In addition the procedures were performed under local anesthesia and well tolerated by the patients that were also superiority than the conventional transnasal approach. Furthermore more accurate positioning of the needle prevents the risk of ocular complications such as diplopia as well as blindness.

SPG block is a safe and easy method for the control of acute or chronic pain than the other percutaneous treatments for TN management even in office settings. It takes only a few moments to implement, and the patient can be safely taught to effectively perform this pain control procedure at office settings with good expectations and results. Opioids are one of the most effective classes of medications available to treat both acute and chronic pain. Opioids act to suppress pain through mu-receptor activation on priary afferent nerve fibers, dorsal horn neurons and supraspinal pain center neurons (Sindrup and Jensen 2002). It has been used effectively in the management of temporomandibular joint (TMJ) pain, cluster headaches, tic douloureux, dysmenorrhea, trigeminal neuralgia, bronchospasm and chronic hiccup (Peterson et al. 1995; Davis and Dostrovsky 1988a, b). Therefore we carried out our study with opiod usage for the SPG blockade that increased success in the patient’s medical treatment. Although SPG blockade with bupivacaine injection has been performed and evaluated for many other pain syndromes, the combination of fentanyl and bupivacaine was found to be an effective way on the management of TN type II. On the other hand effectiveness of bupivacaine alone and mixture of fentanyl with bupivacaine should been compared in with larger study groups. In addition evaluation of the average distance from greater palatine foramen to the SPG and average angulation of the needle may be beneficial for performing the procedures in clinical settings.

As a conclusion, SPG blockade improves the quality of life of patients, though the exact treatment compared to other methods. The method is minimally invasive, even though it may be done in hospital conditions. The complications during the procedure can be tolerated than the other methods are still an advantage for SPG. Further clinical studies are recommended with larger groups.
